# Inter-Dental Splints

**Published:** 1891-02

**Authors:** Alonzo P. Beale

**Affiliations:** Philadelphia


### International Dental Journal.

VOL. XII. FEBRUARY, 1891. No. 2.

### Original Communications.[1]

### INTER-DENTAL SPLINTS.[2]

BY ALONZO P. BEALE, D.D.S., PHILADELPHIA.

IT affords me great pleasure to be with you on this occasion, and to be honored with an invitation to read an essay before the Society.

The title of my paper, as you have observed, is "Inter-Dental Splints,"—splints to be used in treating cases of fracture of the lower jaw. The subject is not a new one by any means, but is one worthy of our attention for a short time.

It is not my intention to enter into a detailed account of the treatment of fractured jaws, but simply to give a complete description of the mechanical details involved in the manufacture of inter-dental splints.

The mechanical principles are simple, and if thoroughly understood and accurately carried out the result will be successful, but if one detail is faultily executed, no matter how carefully the others are performed, the result will be absolute failure.

It is a fact well established that in treating cases of fracture of

[1] The editor and publishers are not responsible for the views of authors of papers published in this department, nor for any claim to novelty, or otherwise, that may be made by them. No papers will be received for this department that have appeared in any other journal published in the country.

[2] Read at the meeting of the Pennsylvania State Dental Society, July 29, 1890.

the inferior maxilla, without the aid of an inter-dental splint, great difficulty is experienced in keeping the fragments in juxtaposition, especially where there is a double fracture.

Although their use is recommended to students in medical colleges, there are still many cases treated in the old way, with simply a submental splint and a bandage, by surgeons who concern themselves but little as to the future comfort or sightliness of the patient.

As the physician or surgeon cannot make an inter-dental splint, he must of necessity call upon the dentist to make it for him; hence every dentist should be perfectly familiar with all the mechanical details requisite for the formation of such an appliance, this being the more necessary as a special splint must be made for each case.

The advantages of the use of such splints are, first, perfect immobility of the fragments, thus securing the best possible condition for union of the fractured bone. Second, a complete restoration of the teeth to their normal articulation, with a consequent avoidance of disfigurement and impairment of the masticating functions.

Although I am a strong advocate of the use of plaster of Paris for taking impressions of all cases for artificial dentures where it can be employed, I have arrived at the conclusion, by experience, that plaster is not the best material to use in taking impressions of fractured jaws. The proper materials are either modelling compounds or one of the various wax preparations. As a first step, an upper impression should be obtained in the usual manner, using as little material as needed to insure success without causing the patient any unnecessary pain or inconvenience in opening the mouth. When taking the lower impression an assistant should stand back of the patient and hold the lower jaw firmly with both hands, being careful to place his index-finger or fingers on the point or points of fracture to prevent separation of the fragments while the impression-material is being pressed into position. Notwithstanding the care just mentioned, more or less movement will take place, which will be apparent when the cast has been poured and removed from the impression.

From these impressions plaster casts should be obtained which should then be accurately articulated.

This is accomplished by cutting the lower cast apart with a small saw at the place or places indicating the fracture, and trimming enough plaster from the sawed surfaces to make the teeth of the lower antagonize properly with those of the upper cast.

When this has been done, the several parts of the lower cast should be held in contact with the upper one and freshly-mixed plaster placed on the base of the lower to fasten the pieces together. When the plaster has set, place the two casts in an articulator. In order to do this correctly it is necessary to know just how far the natural centrals are from the condyloid processes or hinges of the jaw. This distance varies in different individuals, but the average is about three inches and a half. This space should be measured, and the upper cast should be placed in the articulator so that the centrals will be just that far from the hinge of the articulator. Then place the lower cast in the articulator so that the teeth will antagonize with the upper ones.

If the measurement is not taken and the casts are placed too far forward, it will be seen that, when the splint is finished and placed in the mouth, the molars will strike first and will prevent the other teeth from sinking into their proper places. If the casts are placed too far back, the front teeth will strike first and the back teeth will not fit in their proper positions. If either of these unfortunate mistakes should be made, the splint will be a failure, and it will be necessary to take new impressions and make another one. After the casts have been placed in the articulator, the inter-dental spaces should be partially filled with plaster, and any inter-dental dovetail spaces should be so filled as not to cause any impediment to the proper placing of the splint when finished.

When this has been done, the distance the jaws are to be held apart should be decided upon and the articulator so set as to preserve this distance. This space should be wide enough to allow for the free passage of an ordinary feeding-tube.

The plaster teeth, upper and lower, and the portions of the casts representing the gums, about one-quarter of an inch above the teeth, should be covered with heavy tin-foil (No. 60). The object in doing this is to make the splint on its inner aspect a trifle larger than the natural teeth and gums it is to fit over, and to have the tin, by virtue of its thickness, strip from the rubber in the finishing process and leave its inner surface perfectly smooth and finished. After the foil has been burnished to the casts the splint should be modelled in wax. The method preferred is to soften some wax, preferably a mixture of paraffin and wax, and roll it out into sheets about one-twelfth of an inch in thickness. Carefully press this wax over the tin-foil covering of the casts and cut the edges of the wax off a trifle short of the tin so that the foil will extend beyond the wax all around its entire margin. When the upper and lower base-plates have been made, they should be united with a strip of wax a little more than one-eighth of an inch in thickness, wide enough to fit between the upper and lower wax when the articulator is brought together, and extending as far back as they do. The joints between this strip of wax and the base-plates should be carefully filled with melted wax, and the entire piece nicely smoothed with a spatula. The object in having the tin-foil extend beyond the wax is to have the plaster investment in the flask hold it firmly in position so that it will not become displaced while the rubber is being packed. The casts should be removed from the articulator and their bases pared down so that both casts, with wax in position, can be easily placed in the vulcanizing flask. For this purpose a large, deep flask is requisite.

In flasking, partially fill the lower section with plaster and place the lower cast in it and allow the plaster to cover just half of the wax, thus making the line of separation of the sections of the flask just in the middle of the wax-union between the base-plates. When the plaster has been made smooth, it should be soaped and the flasking finished in the usual manner. When the plaster has hardened, the flask should be placed in warm water and allowed to remain there long enough to soften the wax sufficiently so that the edges of the plaster investment will not be fractured on opening the flask. When the flask is parted, small surplus gates should be made; then the wax should be washed out with boiling water. The wax should not be picked out with instruments, as by so doing the foil might be displaced and the casts broken. When the wax has been removed and the moulds dried, the rubber should be packed, using thin strips and delicate instruments to insure forcing the rubber into every part of the mould. When the moulds are full, place the sections together and the bolts in their places, close the flask, and then vulcanize. If the waxing has been done carefully and neatly, and the piece made as thin as permissible for strength, the vulcanizing can be done precisely as in ordinary cases. When the vulcanizing process is completed and the flask cold, the splint should be taken out of the flask and the plaster removed from it. The tin-foil should be stripped from the rubber and then the finishing should be done in the usual way employed for finishing rubber plates. The double advantage of the heavy tin-foil will be apparent at this stage of the operation. When it is removed from the splint the rubber will be perfectly smooth and also a trifle larger than the parts it is to fit over, which is a decided advantage, as the splint will pass into position easily and will not require fitting in the final adjustment of the teeth to the splint. There is another method of accomplishing the same result which saves time and work, but which requires decidedly more skill.

After having placed the casts in an articulator and covered the proper portions of them with tin-foil, instead of making the wax form as described, the splint can be made at once of rubber.

First paint the tin-foil with liquid rubber about the consistency of cream, and then place rubber on in suitable strips to form the splint in a manner similar to that pursued in making the wax one, being careful to press the rubber firmly against the models in order to make it stick to the rubber cement. When this has been done, the strip connecting the two should be about two layers of rubber in thickness, and should be nicely fitted between the upper and lower plates and fastened to them. The joints between the rubber can be made smooth by the use of a hot spatula. If care is exercised the rubber can be manipulated and shaped with a hot spatula almost as easily as wax.

When this has been done, the inner space between the upper and lower casts should be filled with plaster before removing them from the articulator. If the casts should be removed from the articulator without this support of plaster between them, the rubber being soft and elastic, the casts might become displaced and the shape and fit of the splint would be ruined.

When the plaster is hard, saw the casts from the articulator and embed the casts and rubber in plaster in the flask; when the investment is hard, proceed with the vulcanizing. The advantage of this method is that it consumes less time than the former one, and the tedious operation of packing the rubber into the small crevices of the mould is done away with.

In the finishing process the opening for feeding purposes and others for cleansing should be made. The one for feeding should be large enough to admit a feeding-tube. If desired, other openings can be made in order to more clearly show when the splint is in its proper position when placed in the mouth.

Splints made in this way are also applicable to fractures of the superior maxillary.

This method of vulcanizing over casts covered with thick tin-foil can be employed in making small vulcanite pieces or caps for correcting irregularities of the teeth, and the piece can be fastened in position by any device requisite for the case in hand.

